# Supplementary material for: Combined Multiplexed Phage Display, High-Throughput Sequencing, and Functional Assays as a Platform for Identifying Modulatory VHHs Targeting the FSHR
Source: Int J Mol Sci. 2023 Nov 4;24(21):15961. doi: 10.3390/ijms242115961 (PMC10650796; doi:10.3390/ijms242115961)
Supplement: Supplementary file 1 [file ijms-24-15961-s001.zip › ijms-2657368-supplementary.pptx]

## Slide 1
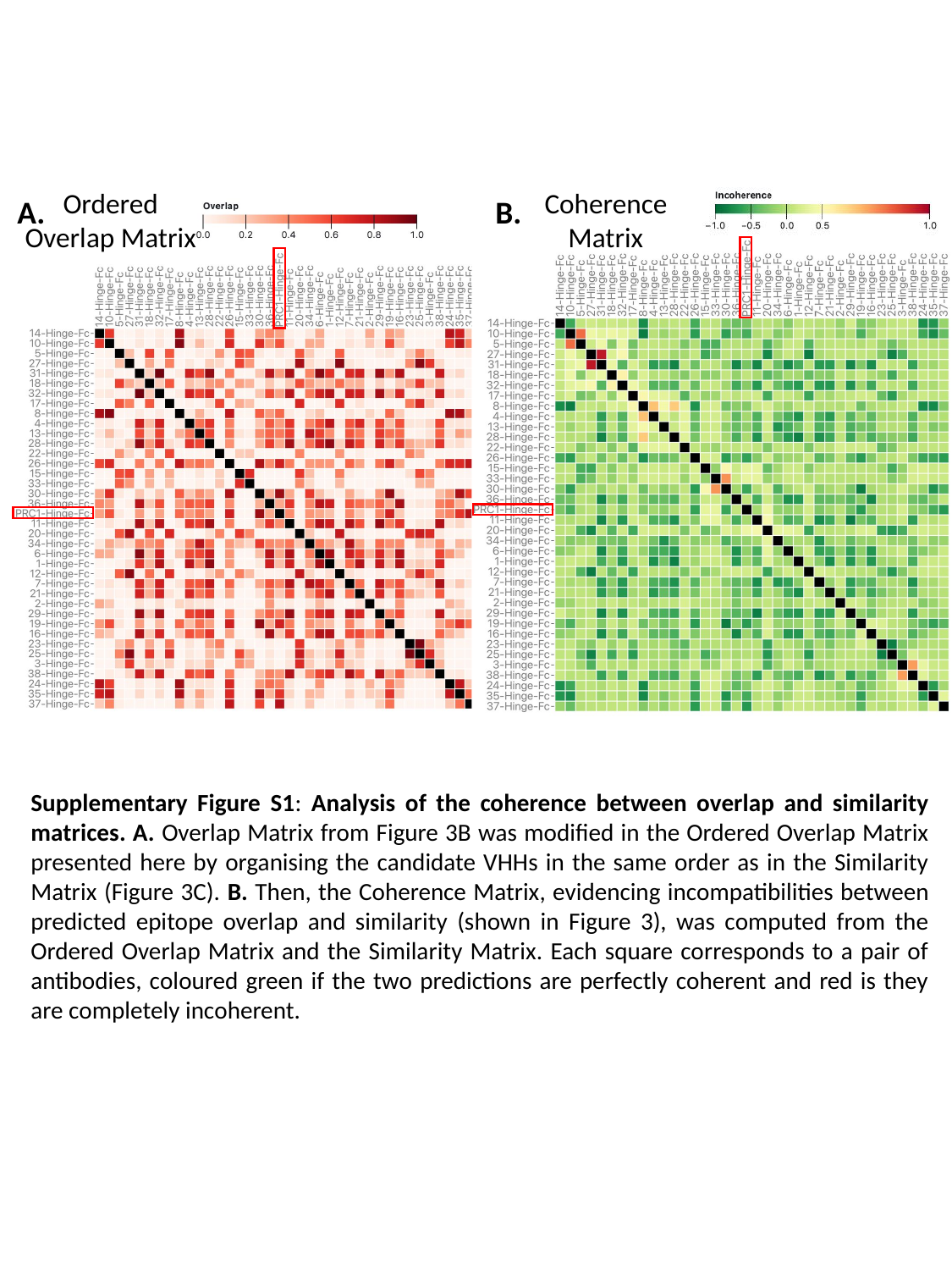

Ordered Overlap Matrix
A.
Coherence Matrix
B.
Supplementary Figure S1: Analysis of the coherence between overlap and similarity matrices. A. Overlap Matrix from Figure 3B was modified in the Ordered Overlap Matrix presented here by organising the candidate VHHs in the same order as in the Similarity Matrix (Figure 3C). B. Then, the Coherence Matrix, evidencing incompatibilities between predicted epitope overlap and similarity (shown in Figure 3), was computed from the Ordered Overlap Matrix and the Similarity Matrix. Each square corresponds to a pair of antibodies, coloured green if the two predictions are perfectly coherent and red is they are completely incoherent.

## Slide 2
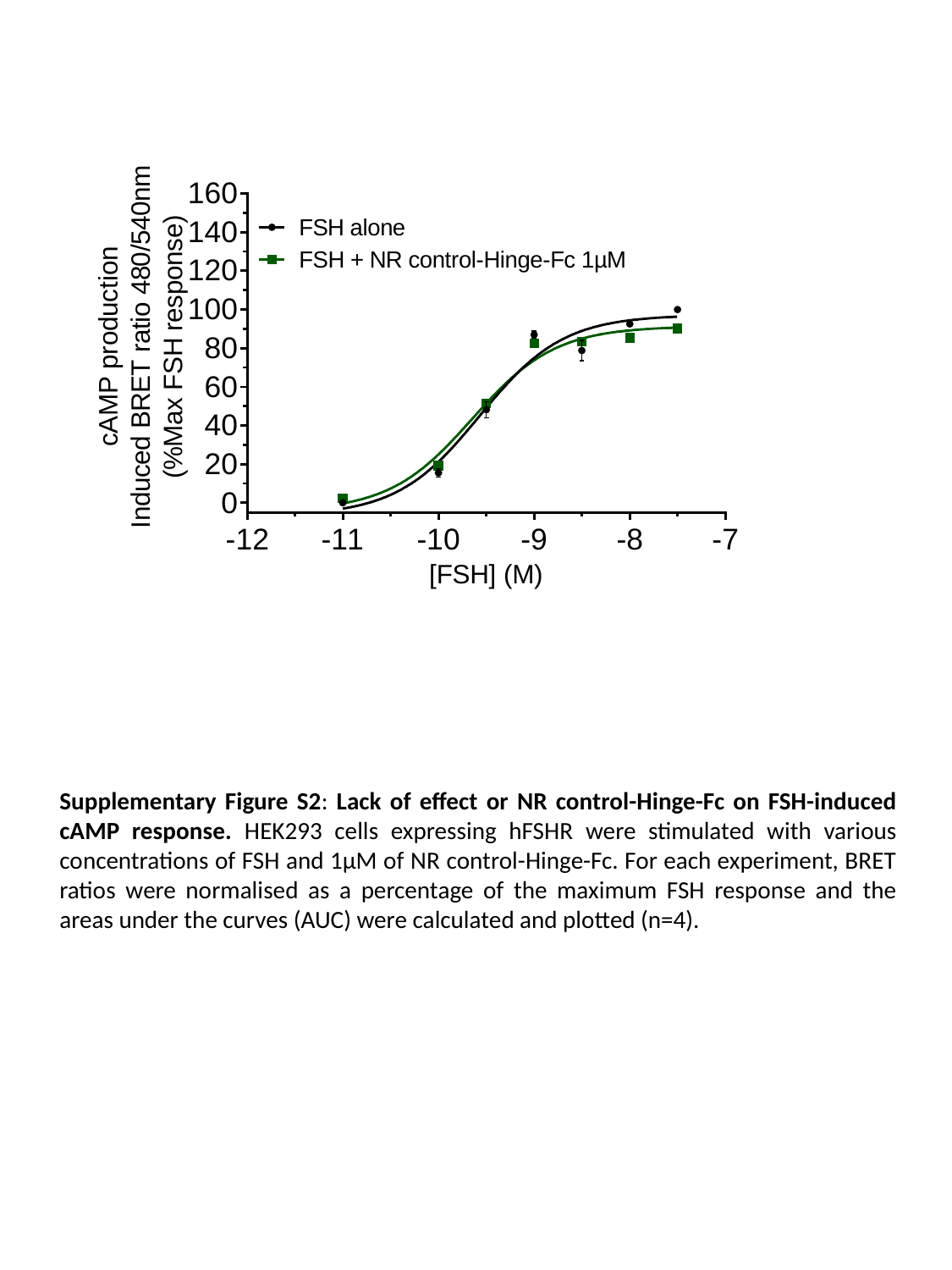

Supplementary Figure S2: Lack of effect or NR control-Hinge-Fc on FSH-induced cAMP response. HEK293 cells expressing hFSHR were stimulated with various concentrations of FSH and 1µM of NR control-Hinge-Fc. For each experiment, BRET ratios were normalised as a percentage of the maximum FSH response and the areas under the curves (AUC) were calculated and plotted (n=4).
